# Measuring what matters: challenges in pain assessment among people with dementia and rheumatic and musculoskeletal diseases

**DOI:** 10.1016/j.ero.2026.01.012

**Published:** 2026-02-22

**Authors:** Shennah Austen, Daisy J.A. Janssen, Annelies Boonen, Astrid van Tubergen, Marloes van Onna

**Affiliations:** 1 Department of Health Services Research, CAPHRI Care and Public Health Research Institute, Faculty of Health, Medicine and Life Sciences, Maastricht University, Maastricht, The Netherlands; 2 Cicero Zorggroep, Zuid-Limburg, Brunssum, The Netherlands; 3 Department of Family Medicine, CAPHRI Care and Public Health Research Institute, Faculty of Health, Medicine and Life Sciences, Maastricht University, Maastricht, The Netherlands; 4 Department of Expertise and Treatment, Proteion, Horn, The Netherlands; 5 Department of Rheumatology, Maastricht University Medical Center, Maastricht, The Netherlands; 6 CAPHRI Care and Public Health Research Institute, Faculty of Health, Medicine and Life Sciences, Maastricht University, Maastricht, The Netherlands

## Abstract

Population ageing will lead to a growing number of people living with both dementia and rheumatic and musculoskeletal diseases (RMDs). Current clinical practice and research methods in rheumatology are, however, largely built around people who can reliably communicate symptoms and report pain. Dementia fundamentally alters how RMDs present, as joint pain may manifest through behavioural change, and treatment response may not be captured by standard assessments such as disease activity scores. Observational pain scales used in geriatric care, such as the Pain Assessment Checklist for Seniors with Limited Ability to Communicate, were developed for routine clinical observation. These tools are, however, not specific to RMD-related symptoms. We argue that pragmatic study designs incorporating outcomes collected in daily living situations, such as mobility and qualitative input from informal caregivers and nursing staff, are needed to better detect treatment effects in people with dementia and RMDs.

## INTRODUCTION

The global population is ageing rapidly. Approximately 1 in 5 Europeans is currently aged 65 years or older, and by 2050, older adults are expected to make up nearly one-third of the total population [1,2]. Population ageing is inevitably accompanied by a higher prevalence of age-related diseases, including both dementia and common rheumatic and musculoskeletal diseases (RMDs) such as osteoarthritis. In Europe, approximately 1.8% of people currently live with dementia—around 11.3 million people—and this proportion is projected to rise to 3% (about 18.8 million people) by 2050 [[3], [4], [5]].

While these demographic trends are important across all medical specialties, their significance for rheumatology is frequently underestimated. As the number of older people with dementia continues to grow, rheumatology professionals will become increasingly involved in the diagnosis and management of RMDs in this vulnerable population [6]. Dementia is a clinical syndrome characterised by a progressive decline in cognitive abilities—such as memory, language, executive function, and orientation—that interferes with daily functioning and social independence [7,8]. People with dementia and RMDs differ fundamentally from those without cognitive impairment: RMDs may present atypically (eg, behavioural change instead of reported joint pain), and responses to pharmacological or non-pharmacological treatments can be altered.

Although many people with both RMDs and dementia reside in nursing homes or other long-term care facilities, rheumatologists will increasingly encounter these challenges in outpatient settings, particularly as people with mild-to-moderate cognitive impairment remain living at home for longer. To date, our clinical practice and research methods have been built almost entirely around people who can reliably communicate symptoms, report pain, and participate in standard assessments. In this Viewpoint, we discuss why our current approaches fall short in people with dementia and RMDs, and we propose ways to better assess RMD pain and treatment response in this population.

## THE CHALLENGE OF ASSESSING RMD-RELATED PAIN AND TREATMENT RESPONSE

Procedures that are routine in rheumatology—such as disease activity scores, tender joint counts, or even a standard physical examination—are often unreliable in people with dementia. Noninflammatory RMDs, such as osteoarthritis, are highly prevalent in people with dementia, whereas inflammatory RMDs may be less common but potentially more prone to under-recognition, as their assessment relies more on symptom reporting and physical examination. While untreated pain may contribute to behavioural symptoms and functional decline, inappropriate initiation of analgesic or anti-inflammatory therapies can aggravate cognitive impairment and increase the risk of other adverse events. Box presents a case that illustrates the challenge of assessing joint pain and evaluating treatment response in people with dementia: despite clear clinical improvement, standard observational pain scales did not detect meaningful change, revealing a gap between *what we observe* and *what we can measure* with currently available tools. This limitation not only affects treatment decisions in clinical practice but also poses a significant challenge in clinical research, where quantifiable outcomes are essential. If we want to improve care for people with dementia and RMDs, we need assessment tools that can capture pain and changes in pain, as a basis for developing and evaluating effective approaches to managing RMD symptoms [1,8].

**Illustrative case**

An 84-year-old wheelchair-bound female nursing home resident with Alzheimer’s disease and Behavioural and Psychological Symptoms of Dementia (claiming, screaming, hallucinations), chronic obstructive pulmonary disease, chronic kidney disease, and osteoarthritis presents with persistent right shoulder pain. The pain is continuous and markedly worsens during care routines, such as dressing, during which she becomes defensive and begins to cry. Paracetamol, topical diclofenac, and physiotherapy are all ineffective. Due to a contraindication for oral nonsteroidal anti-inflammatory drugs and a history of severe delirium on opioid therapy, analgesic options are limited. The elderly care physician contacts her legal representative and, after discussion, initiates a trial of prednisone 5 mg once daily for a suspected flare of shoulder osteoarthritis.

Following initiation, the staff observes clear clinical improvement: pain behaviours, such as crying and screaming, diminish, and the patient no longer resists care. This evident improvement is, however, not captured by formal assessment. Her Mobilization–Observation–Behaviour–Intensity–Dementia (MOBID-2) subscore for musculoskeletal pain changes only minimally (eg, from 7/10 pretreatment to 6/10 during treatment). After a few weeks, prednisone was successfully tapered and discontinued without recurrence of symptoms.

Alt-text: Unlabelled box dummy alt text

## PREVALENCE OF PAIN AND TOOLS FOR ITS ASSESSMENT IN PEOPLE WITH DEMENTIA

To improve the assessment of RMD pain, it is important to first consider the prevalence of (suspected) pain in people with dementia. Most available data about (suspected) pain originate from studies conducted in nursing home residents [[9], [10], [11]]. These studies often did not report what proportion of the included nursing home residents actually had dementia. Pain prevalence rates in nursing home residents vary from approximately 9% to 85% [[9], [10], [11]]. This wide range could be explained by differences in measurement approaches, observer variability, and the type of pain or organ system studied. In a study by our group involving 100 nursing home residents (50% with dementia), (suspected) joint pain was identified in half of the participants and previously unrecognised synovial joint swelling in 10%, suggesting that inflammatory RMDs are often undetected in this setting [12]. Synovial swelling was more frequently observed in residents without dementia, likely reflecting differences in admission indications, as residents without dementia were more often admitted because of severe multimorbidity and high levels of care dependency [12]. Furthermore, it is estimated that 25% of nursing home residents suspected to have pain receive no pain treatment [13]. Residents with dementia are ≈20% are less likely to receive pain treatment than those without [14,15].

In the nursing home setting, the presence and severity of (suspected) pain is assessed through a combination of self-report when possible and observation. Self-report is the cornerstone of pain assessment, as it best reflects the subjective experience of pain [8]. For those with moderate cognitive impairment, the Verbal Descriptor Scale is often preferred by nursing home residents themselves, as it asks residents to rate their pain using simple verbal categories such as ‘no pain’, ‘mild’, ‘moderate’, or ‘severe’ [16].

However, when self-report is not possible, several observational pain scales can be used, including the Pain Assessment Checklist for Seniors with Limited Ability to Communicate (PACSLAC), the Pain Assessment in Advanced Dementia, and the Mobilization–Observation–Behaviour–Intensity–Dementia (MOBID-2) scale (Table) [[17], [18], [19], [20]]. The PACSLAC, for example, consists of a list of pain-related behaviours divided into items, such as facial expressions, body movements, vocalisations, and changes in social interactions and mood [17]. In a study among nursing home residents, use of the PACSLAC identified previously unrecognised pain behaviours in 27% of residents [21]. It remains, however, uncertain whether these findings represent true pain or false-positive identified pain behaviours. The MOBID-2 scale provokes pain through guided movements, such as turning in bed, making it somewhat more relevant in the RMD-setting (Table). It should, however, be noted that these movement-based items provide only a global impression of the presence of musculoskeletal pain but still do not assess joint-specific pathology (eg, synovial swelling, joint tenderness) [20,22].

**Table:** Commonly used observational pain assessment instruments for people with moderate to severe dementia

Instrument	Main domains assessed	Additional features/observation context	Scoring principle
PACSLAC^a^ [17]	• Facial expressions (eg, sad look, frowning) • Activity/body/movement (eg, wandering, resisting care) • Social/personality/mood (eg, physical or verbal aggression) • Physiological/other (eg, teary-eyed, appetite changes)	Designed for routine clinical observation. Captures general pain behaviours. In RMDs, movement-related items (eg, resistance during care) may be most informative.	Original version 60 items; each rated present/absent; a higher total indicates more pain
PAINAD [18]	• Breathing (eg, noisy) • Negative vocalisations (eg, groaning) • Facial expression • Body language (eg, fidgeting, distressed) • Consolability	Developed for people with advanced dementia, routine clinical observation. In RMDs, increases during movement may suggest musculoskeletal pain.	5 items, each scored 0-2; total score 0-10; higher scores indicate more pain
MOBID/MOBID-2 [19,20]	Five guided movements designed to provoke musculoskeletal pain while observing pain-related behaviours: • Open both hands, one hand at a time • Stretch both arms towards head, one arm at a time • Stretch and bend both knees and hips, one leg at a time • Turn in bed to both sides • Sit at the bedside MOBID-2 adds assessment of pain indicators from internal organs, head and skin (visceral, neuropathic, dermatologic).	Designed for routine clinical observation, responsive to treatment; clinically relevant change ≥3 points More relevant for RMDs than other tools: structured guided movements facilitate detection of movement-evoked joint pain, although scores remain non–joint-specific.	MOBID-2 is an expanded version of MOBID, also including nonmusculoskeletal pain sources, such as visceral and skin pain. Items scored 0-10; total 0-50 (MOBID)/0-100 (MOBID-2); higher scores indicate more pain

MOBID, Mobilization–Observation–Behaviour–Intensity–Dementia; PACSLAC, Pain Assessment Checklist for Seniors with Limited Ability to Communicate; PAINAID, Pain Assessment in Advanced Dementia; RMD, rheumatic and musculoskeletal disease.

a Different versions are available that differ in the number of domains and items that are included.

## IMPORTANCE OF CONTEXT

In a study comparing PACSLAC scores rated by nurses or independent observers over a 3-month period in nursing home residents with severe dementia, the independent observers’ ratings showed no change over time. In contrast, the nurse-obtained scores decreased, most likely because higher scores led to additional pain medication being given [23]. Although it is difficult to determine which perspective was more accurate, it is possible that the independent observers failed to detect subtle behavioural improvements because their observation periods were brief, whereas nurses continuously observed residents in daily care and potentially started or increased pain medication. Pain scores derived by independent observers were lower than those obtained by nursing staff [23]. These findings highlight that assessing pain in people with dementia requires instruments that reflect how pain actually occurs in daily life. In the case of RMDs, this can be challenging as musculoskeletal pain is often intermittent and triggered by specific tasks, rather than constantly present. As a result, RMD pain may not produce a clear or recognisable behavioural pain pattern in people with dementia. It is therefore important to consider contextual factors such as the activity during which (suspected) pain emerges (eg, during transfers), using sufficiently long and repeated observations to capture variability in pain behaviours and the observer’s familiarity with the resident’s baseline behaviour. Without these elements, clinically meaningful changes in RMD pain are unlikely to be detected or interpreted accurately.

## RETHINKING TRIAL DESIGN FOR PEOPLE WITH DEMENTIA AND RMDS

The challenges described above arise in clinical care, but they have equally important implications for research. If joint pain and pain severity in people with dementia can only be meaningfully understood through context, repeated observation, and input from those who know the person well, then traditional trial methodologies—which heavily rely on standardised assessments—are unlikely to capture treatment effects accurately. Observational pain scales, such as the PACSLAC, were developed for everyday clinical practice rather than for research settings. They are neither specific to RMD pain nor validated for assessing treatment response in this population. Future RMD studies should move beyond highly controlled randomised trials and adopt more pragmatic approaches [24]. Such approaches could include outcomes that are naturally generated within daily clinical practice—such as mobility, participation in daily activities, and signs of pain behaviour. In addition, qualitative or mixed-methods designs may help capture treatment effects that are not reflected in routine data. Semistructured interviews or focus groups with informal caregivers and nursing staff could provide additional insights regarding contextual factors. For instance, in the case presented in the Box, had this been a clinical trial, interviews with nursing staff and family members could capture perceived changes in the resident’s comfort during morning care or mobilisation.

A further consideration, often unfamiliar to rheumatology researchers, is the need to conduct study procedures in participants who may show resistance or protest. In people with dementia, behaviours such as pulling away or saying ‘no’ may reflect a known behavioural pattern or a pain response rather than ‘true’ resistance. Therefore, trials should include someone who knows the participant well and can distinguish pain-related or ‘normal’ behaviours from genuine protest.

## CONCLUSION

Dementia fundamentally alters how RMD pain can be assessed and treatment effects can be measured. Observational pain scales used in geriatrics often fail in people with RMDs and dementia. Pragmatic study designs incorporating innovative instruments collected in the daily living situation and qualitative input from informal caregivers and nursing staff are needed to detect treatment effects that standard RMD instruments fail to capture.

## CRediT authorship contribution statement

**Shennah Austen:** Writing – review & editing, Writing – original draft. **Daisy J.A. Janssen:** Writing – review & editing, Writing – original draft. **Annelies Boonen:** Writing – review & editing, Writing – original draft. **Astrid van Tubergen:** Writing – review & editing, Writing – original draft. **Marloes van Onna:** Writing – review & editing, Writing – original draft, Conceptualization.

## Competing interests

DJAJ has received research grants within the past 36 months from the Netherlands Organisation for Health Research and Development, the European Union’s Horizon Europe research and innovation programme, and Proteion, all outside the submitted work and all paid to the institution. AB received research grants from AbbVie and Eli Lilly and honoraria for lectures or consultation from Pfizer, UCB, AbbVie, and Alfasigma; all to her department. AvT received unrestricted research grants from Novartis and UCB, received consulting fees from AbbVie, Novartis, Johnson and Johnson, and UCB and received speakers’ fees from Pfizer and Novartis. MvO has received consultancy fees to her department from Pfizer and Alfasigma, and a research grant from Pfizer, all paid to the institution. The other author has not declared consultancy fees or a specific grant for this research from any funding agency in the public, commercial or not-for-profit sectors.
